# Penicillin allergy labels increase risks of MRSA colonization, surgical site infections, and mortality

**DOI:** 10.1111/pai.70378

**Published:** 2026-06-08

**Authors:** Heejo Keum, Juhee Lee, Santiago Alvarez‐Arango, Timothy G. Chow

**Affiliations:** ^1^ Department of Internal Medicine University of Texas Southwestern Medical Center Dallas Texas USA; ^2^ Division of Allergy and Immunology Children's Hospital of Philadelphia Philadelphia Pennsylvania USA; ^3^ Division of Allergy and Immunology University of Texas Southwestern Medical Center Dallas Texas USA

**Keywords:** antimicrobial stewardship, mortality, MRSA colonization, penicillin allergy label, surgical site infection

**Abbreviations**
CI: confidence interval
DM: diabetes mellitus
EHR: electronic health record
ICD‐10‐CM: International Classification of Diseases, Tenth Revision, Clinical Modification
ICD‐9‐CM: International Classification of Diseases, Nineth Revision, Clinical Modification
MRSA: methicillin‐resistant *Staphylococcus aureus*
PAL: penicillin allergy label
RR: relative risk
SCD: sickle‐cell disorder
SD: standard deviation
SSI: surgical site infection

To the Editor,

Penicillin allergy labels (PAL) are the most documented drug allergy in electronic health records (EHR).^1^ In a large birth cohort from 90 hospital‐affiliated pediatric primary care practices, PAL prevalence was estimated at 5.4%.^2^ Although most children labeled as penicillin‐allergic are not truly allergic, PALs often lead to avoidance of first‐line β‐lactams. In adults, such avoidance has been associated with increased methicillin‐resistant *Staphylococcus aureus* (MRSA) colonization, surgical site infections (SSI), and all‐cause mortality.^3^, ^4^ However, pediatric data remain limited. Existing pediatric studies are largely single‐center and underpowered.^5^ A recent population‐based study of pediatric patients with community‐acquired pneumonia linked PALs to increased risk of hospitalization, acute respiratory failure, and need for intensive care use.^6^ Other adverse health outcomes, specifically MRSA colonization, surgical site infections, and all‐cause mortality, remain unknown in pediatric populations. This study evaluated MRSA colonization, SSI, and all‐cause mortality among pediatric patients with PAL.

This retrospective matched cohort study used data extracted on October 20, 2025 from the TriNetX US Collaborative Network, which includes EHRs from approximately 123 million patients across 68 healthcare organizations. Eligible participants were younger than 18 years at the index visit, defined as the first encounter between January 1, 2007 and January 1, 2017. Inclusion required at least one visit during the index period and one subsequent visit between January 1, 2017 and December 31, 2024 to ensure longitudinal follow‐up and outcome ascertainment. Patients with documented MRSA colonization prior to January 1, 2017 were excluded to establish a clear baseline and minimize misclassification of prevalent cases.

Pediatric patients with PALs were identified by International Classification of Diseases, Ninth and Tenth Revisions, Clinical Modification (ICD‐9‐CM and ICD‐10‐CM) codes, including ICD‐10‐CM code Z88.0 (allergy status to penicillin) and/or ICD‐9‐CM code V14.0 (personal history of allergy to penicillin).^6^ Controls were pediatric patients without these codes. Primary outcomes included MRSA colonization, SSI, and all‐cause mortality. MRSA colonization was identified using ICD codes and laboratory values (Table S1). SSI was defined by ICD‐10‐CM code T81.4 (infections following a procedure). All‐cause mortality was determined using the “deceased” status indicator available in TriNetX.

Baseline characteristics were selected a priori based on prior literature and clinical relevance and compared between patients with and without PALs (Table S2). Age and sex were included in all analyses. Additional comorbidities present in at least 1% of both cohorts were considered for propensity score matching. Variables selected for matching included body mass index ≥95th percentile to <120% of the 95th percentile for age, diabetes mellitus (DM), congenital malformations of heart, malformations of cardiac septa, heart failure, sickle‐cell disorder (SCD), and malignant neoplasms of lymphoid, hematopoietic, and related tissue, given their potential to confound infection and mortality outcomes.^7^ The number of variables included in the propensity score model was limited to 10 due to platform constraints within the TriNetX.

All statistical analyses were performed in real time using the TriNetX analytics platform. We used the Pearson *χ* ^2^ test for categorical variables and the independent samples *t*‐test for continuous variables. For each patient with PALs, a propensity score was generated and matched with a control patient with a similar propensity score using a nearest‐neighbor approach with a caliper width of 0.1 pooled standard deviations (SDs) of the propensity scores. Patients with PALs and controls were considered well balanced if standardized mean differences were less than 0.1. For each outcome, relative risks (RRs) with 95% confidence intervals (CIs) were calculated. A 2‐sided *p* value <.05 was considered statistically significant. When patient counts were between 1 and 10, TriNetX rounded counts to 10 to protect patient privacy.

A total of 125,802 children with PAL and 9,532,587 children without PAL were identified. After 1:1 propensity score matching, 125,792 patients with PAL were matched to 125,792 controls (Table 1). Baseline characteristics before and after matching are shown in Table S2. Before matching, patients with PAL were older than controls (mean age, 15.6 vs. 13.1 years) and were more likely to be female and to have obesity, DM, congenital malformations of the heart, SCD, malignancy, and heart failure. After matching, standardized mean differences for these characteristics were lower than 0.001, indicating excellent covariate balance between groups.

**TABLE 1:** Negative health outcomes in pediatric patients with penicillin allergy label compared to controls after propensity score matching.

Outcome	PAL (*n* = 125,792)	No PAL (*n* = 125,792)	Absolute risk difference, %^a^	RR (95% CI)^b^	*p* value
MRSA colonization	2039 (1.6)	794 (0.6)	+1.0	2.57 (95% CI, 2.37–2.79)	<0.001
SSI	923 (0.7)	299 (0.2)	+0.5	3.09 (95% CI, 2.71–3.52)	<0.001
All‐cause mortality	782 (0.6)	439 (0.3)	+0.3	1.78 (95% CI, 1.59–2.00)	<0.001

Abbreviations: CI, confidence interval; MRSA, methicillin‐resistant *Staphylococcus aureus*; PAL, penicillin allergy label; RR, relative risk; SSI, surgical site infection.

^a^ Absolute risk difference calculated as PAL minus No PAL.

^b^ Relative risks derived from 1:1 propensity score–matched analysis.

After 1:1 propensity score matching (*n* = 125,792 per group), MRSA colonization occurred in 2,039 children with PAL (1.6%) compared with 794 controls (0.6%), corresponding to an absolute risk difference of 1.0 percentage point (number needed to harm [NNH]≈100) and a relative risk (RR) of 2.57 (95% CI, 2.37–2.79; *p* < .001) (Table 1). Surgical site infection was observed in 923 children with PAL (0.7%) versus 299 controls (0.2%), an absolute difference of 0.5 percentage points (NNH≈200; RR, 3.09; 95% CI, 2.71–3.52; *p* < .001). All‐cause mortality occurred in 782 children with PAL (0.6%) compared with 439 controls (0.3%), an absolute difference of 0.3 percentage points (NNH≈333; RR, 1.78; 95% CI, 1.59–2.00; *p* < .001).

This matched cohort analysis demonstrates that pediatric patients with PALs have higher risks of MRSA colonization, SSI, and all‐cause mortality after propensity score matching for measured confounders. These findings are consistent with prior observations in adults and extend similar associations to pediatric cohorts.^3^, ^4^, ^8^ Because most PALs are acquired early in life (≤2 years of age) and rarely undergo formal allergy evaluation, many children carry unverified labels throughout childhood and into adulthood.^2^ PALs have been associated with increased use of broad‐spectrum and second‐line antibiotics, antimicrobial resistance, and higher healthcare costs.^9^ In line with these findings, a 10‐year pediatric birth cohort demonstrated higher rates of adverse drug events among children with PALs treated for respiratory infections.^10^ Importantly, PAL represents a potentially modifiable factor, as prior studies indicate that ≥90% of pediatric patients tolerate direct amoxicillin challenge and can have the PAL safely removed.^11^

Strengths of this study include the use of a large, multicenter database capable of evaluating relatively uncommon outcomes. This study was the first study to show the same outcomes as in adult populations, demonstrating that the burden of PALs is not only there for adults but for children as well. Limitations include the retrospective design and reliance on ICD‐9‐CM and ICD‐10‐CM codes, which may introduce misclassification. In addition, the TriNetX platform limited the number of variables included in the propensity score matching; however, excluded comorbidities were infrequent (<1% of the cohort) and unlikely to substantially influence the findings. In conclusion, this matched cohort study observed that children with PALs are at increased risk of MRSA colonization, SSI, and all‐cause mortality. These findings highlight additional adverse outcomes associated with PAL in the pediatric population and draw attention to PAL as a potential modifiable risk factor that may be addressed to mitigate adverse health outcomes associated with unnecessary penicillin avoidance.

## AUTHOR CONTRIBUTIONS

**Juhee Lee:** Conceptualization; methodology; investigation; validation; supervision; writing – review and editing. **Santiago Alvarez‐Arango:** Writing – review and editing; conceptualization; methodology; investigation; validation; supervision. **Timothy G. Chow:** Conceptualization; investigation; methodology; validation; visualization; writing – review and editing; project administration; supervision; resources; data curation. **Heejo Keum:** Conceptualization; methodology; software; data curation; investigation; validation; formal analysis; visualization; project administration; resources; writing – original draft; writing – review and editing.

## CONFLICT OF INTEREST STATEMENT

T.G. Chow receives grant support from the National Institutes of Health (grant U01 AI184071) unrelated to the present work. S Alvarez‐Arango receives support from the National Institutes of Health (grant UG3AI190386) and through the American Academy of Allergy, Asthma & Immunology (AAAAI) Foundation Faculty Development Award unrelated to the present work. The rest of authors declare that they have no conflict of interest.

## Supporting information


**Table S1:** International Classification of Diseases, Ninth and Tenth Revisions, Clinical Modification (ICD‐9‐CM/ICD‐10‐CM) and Logical Observation Identifiers, Names and Codes (LOINC), and Systematized Nomenclature of Medicine—Clinical Terms (SNOMED CT) used.
**Table S2:** Baseline characteristics of patients with PAL and controls before and after Propensity score matching.

## Data Availability

The data that support the findings of this study are available from TriNetX. Restrictions apply to the availability of these data, which were used under license for this study. Data are available from the author(s) with the permission of TriNetX.
