# Supplementary material for: Penicillin allergy labels increase risks of MRSA colonization, surgical site infections, and mortality
Source: Pediatr Allergy Immunol. 2026 Jun 8;37(6):e70378. doi: 10.1111/pai.70378 (PMC13244409; doi:10.1111/pai.70378)
Supplement: Supplementary file 1 — Table S1: International Classification of Diseases, Ninth and Tenth Revisions, Clinical Modification (ICD‐9‐CM/ICD‐10‐CM) and Logical Observation Identifiers, Names and Codes (LOINC), and Systematized Nomenclature of Medicine—Clinical Terms (SNOMED CT) used. Table S2: Baseline characteristics of patients with PAL and controls before and after Propensity score matching. [file PAI-37-e70378-s001.docx]

**SUPPLEMENTARY TABLES**

| **Supplementary Table 1.** International Classification of Diseases, Ninth and Tenth Revisions, Clinical Modification (ICD-9-CM/ICD-10-CM) and Logical Observation Identifiers, Names and Codes (LOINC), and Systematized Nomenclature of Medicine—Clinical Terms (SNOMED CT) Used. | |
| --- | --- |
| **Outcome or Covariate** | **Codes (ICD-9-CM/ICD-10-CM, LOINC, SNOMED CT)** |
| **PAL** | **Z88.0** (allergy status to penicillin)**; V14.0** (personal history of allergy to penicillin) |
| **MRSA colonization** | **B95.62** (MRSA infection as the cause of diseases classified elsewhere)  **A49.02** (MRSA infection)  **J15.212** (Pneumonia due to MRSA)  **Z22.322** (Carrier or suspected carrier of MRSA)  **Z86.14** (Personal history of MRSA)  **72892-3** (MRSA SCCmec and mecA genes panel - Nose by NAA with probe detection)  **86622-8** (MRSA SCCmec and mecA+mecC genes panel - Nose)  **35492-8** (MRSA DNA [Presence] in Specimen by NAA with probe detection)  **13317-3** (MRSA [Presence] in Specimen by Organism specific culture)  **52969-3** (MRSA [Presence] in Nose by Organism specific culture)  **90001-9** (MRSA DNA [Presence] in Nose by NAA with probe detection)  **77682-3** (MRSA SCCmec+orfX junction [Presence] in Nose by NAA with probe detection)  **94512-1** (Staphylococcus aureus and MRSA identified in Isolate or Specimen by Molecular genetics method) |
| **SSI** | **T81.4** (infections following a procedure​) |
| **BMI** | **Z68.53** (BMI pediatric, 85th percentile to less than 95th percentile for age)  **Z68.54** (BMI pediatric, 95th percentile for age to less than 120% of the 95th percentile for age  **Z68.55** (BMI pediatric, 120% of the 95th percentile for age to less than 140% of the 95th percentile for age)  **Z68.56** (BMI pediatric, greater than or equal to 140% of the 95th percentile for age) |
| **Obesity class** | **E66.81** |
| **DM** | **E08-E13** |
| **Fibrosis and cirrhosis of liver** | **K74** |
| **CM of aortic and mitral valves** | **Q23** |
| **CM of pulmonary and tricuspid valves** | **Q22** |
| **CM of cardiac septa** | **Q21** |
| **CM of cardiac chambers and connections** | **Q20** |
| **Other CM of heart** | **Q24** |
| **CM of great arteries** | **Q25** |
| **HF** | **I50** |
| **SCD** | **D57** |
| **Malignant neoplasms of lymphoid, hematopoietic and related tissue** | **C81-C96** |
| **Asplenia (congenital)** | **Q89.01** |
| **Combined immunodeficiencies** | **D81** |
| **Immunodeficiency with predominantly antibody defects** | **D80** |
| **Bone marrow transplant status** | **Z94.81** |
| **Stem cells transplant status** | **Z94.84** |
| **Solid organ transplant** | **313039003** |
| **HIV** | **B20** |
| **Asthma** | **J45** |
| **Bronchiectasis** | **J47** |
| **CF** | **E84** |
| **Nephrotic syndrome** | **N04** |
| **CKD3** | **N18.3** |
| **CKD4** | **N18.4** |
| **CKD5** | **N18.5** |
| **ESRD** | **N18.6** |
| Abbreviations: BMI, body mass index; CF, cystic fibrosis; CKD, chronic kidney disease; CM, congenital malformations; DM, diabetes mellitus; HF, heart failure; HIV, human immunodeficiency virus; ICD-9-CM, International Classification of Diseases, Ninth Revision, Clinical Modification; ICD-10-CM, International Classification of Diseases, Tenth Revision, Clinical Modification; LOINC, Logical Observation Identifiers, Names and Codes; MRSA, methicillin-resistant *Staphylococcus aureus*; NAA, nucleic acid amplification; PAL, penicillin allergy label; SCD, sickle-cell disease; SSI, surgical-site infection | |

| **Supplementary Table 2.** Baseline Characteristics of Patients with PAL and Controls Before and After Propensity Score Matching | | | | | | | |
| --- | --- | --- | --- | --- | --- | --- | --- |
| Characteristic | Before PSM | | | | After PSM | | |
|  | PAL (125,802) | No PAL (9,532,587) | *p* | SMD† | PAL (125,792) | No PAL (125,792) | SMD† |
| Age at Index | 15.6 (7.8) | 13.1 (7.4) | <0.001 | 0.33 | 15.6 (7.8) | 15.6 (7.8) | <0.001 |
| Female | 71,008 (56.4%) | 4,812,069 (50.5%) | <0.001 | 0.12 | 71,002 (56.4%) | 70,970 (56.4%) | <0.001 |
| Hispanic or Latino | 21,688 (17.2%) | 1,676,818 (17.6%) | <0.001 | 0.01 | 21,685 (17.2%) | 21,492 (17.1%) | 0.00 |
| Black or African American | 18,177 (14.4%) | 1,671,796 (17.5%) | <0.001 | 0.08 | 18,173 (14.4%) | 22,794 (18.1%) | 0.10 |
| Asian | 2,665 (2.1%) | 352,655 (3.7%) | <0.001 | 0.09 | 2,665 (2.1%) | 4496 (3.6%) | 0.09 |
| Metabolic factors |  |  |  |  |  |  |  |
| BMI ≥ 85th percentile to < 95th percentile for age | 3,784 (3%) | 64,723 (0.7%) | <0.001 | 0.17 | 3,782 (3%) | 1,070 (0.8%) | 0.16 |
| BMI ≥ 95th to < 120% of the 95th percentile for age | 5,709 (4.5%) | 114,383 (1.2%) | <0.001 | 0.20 | 5,702 (4.5%) | 5,702 (4.5%) | <0.001 |
| BMI ≥120% to <140% of the 95th percentile for age | 19 (0%) | 37 (0%) | <0.001 | 0.02 | 19 (0%) | 0 (0%) | 0.02 |
| BMI ≥140% of the 95th percentile for age | 22 (0%) | 67 (0%) | <0.001 | 0.02 | 22 (0%) | 10 (0%) | 0.01 |
| Obesity class | 1,367 (1.1%) | 6,580 (0.1%) | <0.001 | 0.13 | 1,366 (1.1%) | 158 (0.1%) | 0.12 |
| DM | 3,068 (2.4%) | 69,723 (0.7%) | <0.001 | 0.14 | 3,061 (2.4%) | 3047 (2.4%) | <0.001 |
| Fibrosis and cirrhosis of liver | 258 (0.2%) | 2,562 (0%) | <0.001 | 0.05 | 258 (0.2%) | 70 (0.1%) | 0.04 |
| Cardiac conditions |  |  |  |  |  |  |  |
| CM of aortic and mitral valves | 1,208 (1%) | 34,705 (0.4%) | <0.001 | 0.07 | 1,205 (1%) | 1,032 (0.8%) | 0.01 |
| CM of pulmonary and tricuspid valves | 845 (0.7%) | 22,590 (0.2%) | <0.001 | 0.06 | 845 (0.7%) | 627 (0.5%) | 0.02 |
| CM of cardiac septa | 2,631 (2.1%) | 101,807 (1.1%) | <0.001 | 0.08 | 2,626 (2.1%) | 2,650 (2.1%) | 0.00 |
| CM of cardiac chambers and connections | 666 (0.5%) | 22,882 (0.2%) | <0.001 | 0.05 | 662 (0.5%) | 901 (0.7%) | 0.02 |
| Other CM of heart | 1,782 (1.4%) | 42,599 (0.5%) | <0.001 | 0.10 | 1,777 (1.4%) | 1,811 (1.4%) | 0.00 |
| CM of great arteries | 1,396 (1.1%) | 51,884 (0.5%) | <0.001 | 0.06 | 1,394 (1.1%) | 1,139 (0.9%) | 0.02 |
| HF | 697 (0.6%) | 11,819 (0.1%) | <0.001 | 0.07 | 688 (0.6%) | 694 (0.6%) | <0.001 |
| Hematologic/oncologic conditions |  |  |  |  |  |  |  |
| SCD | 1,001 (0.8%) | 32,641 (0.3%) | <0.001 | 0.06 | 1,000 (0.8%) | 989 (0.8%) | <0.001 |
| Malignant neoplasms of lymphoid, hematopoietic and related tissue | 859 (0.7%) | 19,434 (0.2%) | <0.001 | 0.07 | 853 (0.7%) | 867 (0.7%) | 0.00 |
| Immunologic conditions |  |  |  |  |  |  |  |
| Asplenia (congenital) | 108 (0.1%) | 1,828 (0%) | <0.001 | 0.03 | 108 (0.1%) | 53 (0%) | 0.02 |
| Combined immunodeficiencies | 74 (0.1%) | 1,259 (0%) | <0.001 | 0.02 | 74 (0.1%) | 21 (0%) | 0.02 |
| Immunodeficiency with predominantly antibody defects | 662 (0.5%) | 8,574 (0.1%) | <0.001 | 0.08 | 662 (0.5%) | 152 (0.1%) | 0.07 |
| Bone marrow transplant status | 182 (0.1%) | 2,440 (0%) | <0.001 | 0.04 | 181 (0.1%) | 82 (0.1%) | 0.02 |
| Stem cells transplant status | 110 (0.1%) | 1,089 (0%) | <0.001 | 0.03 | 109 (0.1%) | 36 (0%) | 0.02 |
| Solid organ transplant | 0 (0%) | 10 (0%) | 0.72 | 0.00 | 0 (0%) | 0 (0%) | Not applicable |
| HIV | 177 (0.1%) | 3,680 (0%) | <0.001 | 0.03 | 177 (0.1%) | 58 (0.1%) | 0.03 |
| Pulmonary conditions |  |  |  |  |  |  |  |
| Asthma | 29,163 (23.2%) | 832,936 (8.7%) | <0.001 | 0.40 | 29,158 (23.2%) | 12,439 (9.9%) | 0.36 |
| Bronchiectasis | 234 (0.2%) | 2,903 (0%) | <0.001 | 0.05 | 234 (0.2%) | 67 (0.1%) | 0.04 |
| CF | 338 (0.3%) | 6,896 (0.1%) | <0.001 | 0.05 | 337 (0.3%) | 111 (0.1%) | 0.04 |
| Renal conditions |  |  |  |  |  |  |  |
| Nephrotic syndrome | 232 (0.2%) | 5,139 (0.1%) | <0.001 | 0.04 | 230 (0.2%) | 93 (0.1%) | 0.03 |
| CKD3 | 269 (0.2%) | 3,917 (0%) | <0.001 | 0.05 | 267 (0.2%) | 109 (0.1%) | 0.03 |
| CKD4 | 171 (0.1%) | 1,942 (0%) | <0.001 | 0.04 | 168 (0.1%) | 62 (0.1%) | 0.03 |
| CKD5 | 148 (0.1%) | 1,538 (0%) | <0.001 | 0.04 | 145 (0.1%) | 51 (0%) | 0.03 |
| ESRD | 330 (0.3%) | 3,717 (0%) | <0.001 | 0.06 | 327 (0.3%) | 102 (0.1%) | 0.04 |
| †: Standardized mean difference (SMD) between two groups less than 0.1 is considered well-balanced.  Abbreviations: BMI, body mass index; CF, cystic fibrosis; CKD, chronic kidney disease; CM, congenital malformation; DM, diabetes mellitus; ESRD, end-stage renal disease; HF, heart failure; HIV, human immunodeficiency virus; PAL, penicillin allergy label; PSM, propensity score matching; SMD, standardized mean difference | | | | | | | |
